# Bacteriological survey of bulk tank milk from dairy farms in Montero, Santa Cruz, Bolivia

**DOI:** 10.14202/vetworld.2018.1506-1509

**Published:** 2018-10-26

**Authors:** Yu Michimuko-Nagahara, Yu Tamura, Masateru Koiwa

**Affiliations:** 1 Japan International Cooperation Agency Volunteers, Montero, Santa Cruz, Bolivia; 2 Department of Immunogenetics, Institute of Tropical Medicine, Nagasaki University, Nagasaki, Japan; 3 Department of Veterinary Clinical Sciences, School of Veterinary Medicine, Rakuno Gakuen University, Ebetsu, Hokkaido, Japan; 4 Veterinary Teaching Hospital, Azabu University, Sagamihara, Kanagawa, Japan.

**Keywords:** bacterial survey, Bolivia, bulk tank milk, dairy farm, mastitis

## Abstract

**Background and Aim:**

Recently, bacterial surveys for mastitis-causing pathogens in bulk tank milk (BTM) have been conducted in several countries worldwide. However, no such surveys have been reported from Bolivia. Therefore, the present study aimed to estimate the prevalence of mastitis pathogens in BTM from dairy farms in Montero, Santa Cruz, Bolivia.

**Materials and Methods:**

Between July 2016 and August 2017, a total of 43 BTM samples were collected from 3264 cows to determine bulk tank somatic cell counts (BTSCC) and identify mastitis-causing bacteria. BTSCC was classified as follows: <100×10^3^, 100-500×10^3^, 500-1000×10^3^, and >1000×10^3^ cells/mL.

**Results:**

Mastitis-causing pathogens identified by agar medium cultures included *Bacillus* spp., coagulase-negative staphylococci (CNS), coliforms, *Staphylococcus aureus* (SA), streptococci, and other species. The proportions of BTSCC of <100×10^3^, 200-500×10^3^, 500-1000×10^3^, and >1000×10^3^ cells/ml were 0%, 37%, 51%, and 12%, respectively. The proportions of coliforms, streptococci, CNS, *Bacillus* spp., SA, and others detected in BTM were 33%, 30%, 16%, 7%, 2%, and 16%, respectively.

**Conclusion:**

Although the herd prevalence of contagious mastitis-causing pathogens, such as SA, in BTM was low, increased BTSCC were identified in Montero, Santa Cruz, Bolivia.

## Introduction

Several studies have estimated the prevalence of major mastitis-causing pathogens in bulk tank milk (BTM) and surveyed bulk tank somatic cell counts (BTSCC) worldwide [1-11]. Assessing the bacterial counts of mammary pathogens in BTM and BTSCC samples have been reported as a useful monitoring tool for identifying major mastitis-causing pathogens in dairy herds [2,6,9]. In particular, *Staphylococcus aureus* (SA) is the most important mastitis-causing contagious bacteria in BTM [4,12]. Moreover, payment programs based on milk quality are used in South America, including Bolivia, as an incentive to improve milk quality [3]. The specific payment program currently implemented in Bolivia is shown in Table-1.

**Table-1:** Additional payment rate based on BTSCC for each class in Bolivia.

Class	BTSCC×10^3^ cells/mL	Payment addition (%)
	0-500	+7
B	500-1000	0
C	1000-2000	−10
C1	2000-3000	−20
C2	>3000	−40

BTSCC=Bulk tank somatic cell count

In Brazil, located next to Bolivia, BTSCC for dairy farms has remained high, with no recent improvement despite the existence of payment programs based on milk quality [3]. High BTSCC was correlated with high levels of contagious mastitis, such as that caused by SA [7]. Moreover, mastitis caused by contagious pathogens is controlled differently than that caused by environmental pathogens [13-16]. Therefore, improving milk quality depends on investigating the association between BTSCC and BTM bacterial infections. However, there are currently no reports on the prevalence of major mastitis-causing pathogens in BTM or on the association between these bacterial infections and BTSCC in Bolivia.

Therefore, the present study aimed to estimate the prevalence of mastitis-causing pathogens in BTM and to determine the association between these pathogens and BTSCC in dairy farms in Montero, Santa Cruz, Bolivia.

## Materials and Methods

### Ethical approval

No live animals were used in the present study. Samples were collected for routine milk testing from BTM by a clinical veterinarian. Therefore, no ethical approval was needed for the current study.

### Study area

The study was conducted in Montero city, which is located to the East of Bolivia and 50 km North of Santa Cruz de la Sierra, the second city of Bolivia. The city is located at 17.3433 S’latitude, 63.2556 W’longitude, and 300 m altitude.

### BTM sample collection

BTM samples were collected from 48 randomly selected dairy herds from July 2016 to August 2017 and used to determine BTSCC and identify mastitis-causing bacteria. Five samples were excluded due to missing BTSCC data. Therefore, 43 samples from 3264 cows were included in the study. Cow breeds included Holstein-Friesian, Brown Swiss, Jersey, Gyr (Gir), and respective mixes.

Sampling was performed according to a standardized protocol. Briefly, BTM was mixed thoroughly for a minimum of 5 min before collection, following which a sample was taken from the top of the bulk tank using gloves and a sterile disposable plastic syringe.

### Bacterial isolation and identification of mastitis-causing pathogens

Mastitis-causing bacteria identified using agar medium cultures included *Bacillus* spp., coliforms, staphylococci, streptococci, and other species. Briefly, a loopful of milk sample (approximately 10 µL) collected from each bulk tank was inoculated onto Trypticase soy agar (Hardy Diagnostics, Santa Maria, CA, USA) enriched with 5% defibrinated sheep blood and MacConkey agar (Hardy Diagnostics). The inoculated plates were then incubated under aerobic conditions at 37°C for up to 72 h and examination of growth, morphology, and hemolytic features, Gram staining, catalase test, and coagulase test were performed.

*Bacillus* spp. were identified based on growth characteristics, hemolysis type, and catalase test positivity from Gram-positive Streptobacilli (rod-shaped). Coliforms were identified based on growth characteristics and lactose fermentation on MacConkey agar from Gram-negative rods. Staphylococci were identified based on positive catalase reaction, hemolysis type, and growth characteristics from Gram-positive cocci. Specific identification of staphylococci was performed using the tube coagulase test (Eiken Chemical, Tokyo, Japan). Streptococci were identified based on negative catalase reaction and growth characteristics. To differentiate between enterococci, catalase-negative and Gram-positive cocci were tested on *Streptococcus faecalis* (SF) media. The inoculated SF test tube was incubated under aerobic conditions at 37°C for 24–48 h [6,17-20]. Unidentified organisms were recorded as other bacteria. The growth characteristics and hemolysis type of each bacterium are shown in Table-2.

**Table-2:** Growth characteristics and hemolysis type of each bacterium for agar culture identification.

Bacteria	Colony size	Colony color	Growth time (days)	Hemolysis type
*Bacillus* spp.	Moderate to large	Yellow	1	β
Coliforms	Large	Gray, Grayness	0.5-1	-
CNS	Moderate	Yellow, Gray, White	1	α, γ
SA	Moderate	Yellow, Gray, White	1	α, αβ, β
Streptococci	Small	Gray, Transparent	1	-

CNS=Coagulase-negative staphylococci, SA=*Staphylococcus aureus*

## Results

### BTSCC proportions

The proportions of BTSCC for <100×10^3^, 100-500×10^3^, 500-1000×10^3^, and >1000×10^3^ cells/mL were 0%, 37%, 51%, and 12%, respectively (Table-3). According to the classification used in Bolivia, the proportion of Class A was 37%, Class B was 51%, Class C was 12%, and Class C1 and C2 were 0%, respectively.

**Table-3:** Proportion and number of BTSCC.

BTSCC×10^3^ cells/ml	Number of samples (tanks)	Proportion of samples (%)	Detected bacteria (number of tanks)
<100	0	0	0
100-500	16	37	Coliforms (6)
			Streptococci (OS) (6)
			Others (2)
			CNS (1)
500-1000	22	51	Coliforms (8)
			CNS (7)
			Others (4)
			Streptococci (OS) (4)
			*Bacillus* spp.(2)
>1000	5	12	Streptococci (OS) (3)
			Others (2)
			SA (1)

BTSCC=Bulk tank somatic cell count, OS=Other streptococci, CNS=Coagulase-negative staphylococci, SA=*Staphylococcus aureus*

### Prevalence of mastitis-causing pathogens in BTM

The prevalence of coliforms, streptococci, coagulase-negative staphylococci (CNS), *Bacillus* spp., SA, and others in BTM was 33%, 30%, 16%, 7%, 2%, and 16%, respectively (Table-4). Coliforms, CNS, streptococci, and others were detected in Class A; *Bacillus* spp., coliforms, CNS, streptococci, and others were detected in Class B; and SA, streptococci, and others were detected in Class C, respectively (Table-3).

**Table-4:** Proportion of bovine mastitis-causing pathogens in 43 BTM samples.

Bacterial growth	Number of samples (tanks)	Proportion of samples (%)
Coliforms	14	33
Streptococci (OS)	13	30
CNS	7	16
*Bacillus* spp.	3	7
SA	1	2
Others	7	16
NG	3	7

BTM=Bulk tank milk, OS=Other streptococci, CNS=Coagulase-negative staphylococci, SA=*Staphylococcus aureus*, NG=No growth

The streptococci identified in this study were not *Streptococcus agalactiae* but other streptococci because beta-hemolysis was not detected in all streptococci (Tables-2-4).

## Discussion

BTM analysis is a useful monitoring tool, which has been used for many years to assess milk quality and screen mastitis-causing pathogens, mainly because the sample analysis is less expensive, more convenient, and faster than testing milk samples from individual animals or groups [1,2,6,9, 21-23].

In the present study, 37% of BTSCC were classified as Class A. However, there were no farms with BTSCC of <100×10^3^ cells/mL, which is the level recognized as no risk of milk yield loss [24,25]. Therefore, increasing the rate of obtaining BTSCC of <100×10^3^ cells/mL classified as Class A is the main strategy to improve BTM quality in Bolivia. Furthermore, 51% of BTM was classified as Class B, which is not associated with an additional payment. Therefore, increasing the rate of obtaining Class A is important for the farmers to increase their gain for the same milk yield. Although Class C BTM represented a proportion of 12%, the fact that it included SA, a contagious pathogen, represents a problem and highlights the need for therapeutic countermeasures against SA for improving BTM quality and preventing the spread of infection in Montero.

In the present study, only Class C BTM presented contagious SA. However, other contagious pathogens, such as *Mycoplasma* spp., could not be excluded from all classes due to methodological limitations. Although SA is reported as the most common contagious pathogen detected in BTM [6,9], all contagious pathogens should be investigated not only by cultural methods but also by genetic testing. Measures such as incorporated post-milking teat dipping, hygienic milking procedures, and strategic use of antibiotic therapy control the spread of contagious mastitis-causing bacteria [13].

In Classes A and B, most detected bacteria were environmental microorganisms (Table-3), which highlights the need for countermeasures against environmental pathogens as a strategy to decrease BTSCC in Montero. Important aspects in the prevention of environmental mastitis-causing pathogens include udder health maintenance; in particular, the application of foremilking is typically recommended and well established in mastitis control programs [14-16].

The present study had some limitations: Cultural methods in agar medium were used due to maneuvering limitations in lacking of the equipment. In future, we hope to use polymerase chain reaction (PCR) or real-time PCR to detect mastitis-causing pathogens including *Mycoplasma* spp. Moreover, we could not perform the drug susceptibility test due to lacking of the facility; thus, an antibiotic therapeutic strategy for the detected mastitis-causing bacteria could not be established.

## Conclusion

The present study determined the prevalence of mastitis-causing bacteria in BTM and BTSCC in Montero, Santa Cruz, Bolivia. The herd prevalence of SA in BTM was low. Further studies using genetic testing, such as PCR and drug susceptibility test for mastitis-causing bacteria in BTM are needed for implementing an adequate therapeutic strategy.

## Authors’ Contributions

YM and YT contributed to the conception and design of the study. YM conducted the sample collection, processing of samples in the laboratory, and participated in the data analysis. MK provided the technical support of the sample analysis. YM and YT prepared the first draft and participated in the correction of the manuscript. YT and MK supervised the research. All the authors have read and approved the final manuscript.

## References

[1] Azevedo C, Pacheco D, Soares L, Romão R, Moitoso M, Maldonado J, Guix R, Simões J (2016). Prevalence of contagious and environmental mastitis-causing bacteria in bulk tank milk and its relationships with milking practices of dairy cattle herds in São Miguel Island (Azores). Trop. Anim. Health Prod.

[2] Bi Y, Wang YJ, Qin Y, Vallverdú RG, García JM, Sun W, Li S, Cao Z (2016). Prevalence of bovine mastitis pathogens in bulk tank milk in China. PLoS One.

[3] Busanello M, de Freitas LN, Winckler J.P.P, Farias HP, Dos Santos Dias CT, Cassoli LD, Machado PF (2017). Month-wise variation and prediction of bulk tank somatic cell count in Brazilian dairy herds and its impact on payment based on milk quality. Ir. Vet. J.

[4] Cortimiglia C, Luini M, Bianchini V, Marzagalli L, Vezzoli F, Avisani D, Bertoletti M, Ianzano A, Franco A, Battisti A (2016). Prevalence of *Staphylococcus aureus* and of *Methicillin-resistant S. aureus* clonal complexes in bulk tank milk from dairy cattle herds in Lombardy region (Northern Italy). Epidemiol. Infect.

[5] DeLong KL, Lambert DM, Schexnayder S, Krawczel P, Fly M, Garkovich L, Oliver S (2017). Farm business and operator variables associated with bulk tank somatic cell count from dairy herds in the Southeastern United States. J. Dairy Sci.

[6] Francoz D, Bergeron L, Nadeau M, Beauchamp G (2012). Prevalence of contagious mastitis pathogens in bulk tank milk in Québec. Can. Vet. J.

[7] Katholm J, Bennedsgaard TW, Koskinen MT, Rattenborg E (2012). Quality of bulk tank milk samples from Danish dairy herds based on real-time polymerase chain reaction identification of mastitis pathogens. J. Dairy Sci.

[8] Norman HD, Lombard JE, Wright JR, Kopral CA, Rodriguez JM, Miller RH (2011). Consequence of alternative standards for bulk tank somatic cell count of dairy herds in the United States. J. Dairy Sci.

[9] Riekerink R.G.O, Barkema HW, Veenstra S, Poole DE, Dingwell RT, Keefe GP (2006). Prevalence of contagious mastitis pathogens in bulk tank milk in Prince Edward Island. Can. Vet. J.

[10] Rysanek D, Zouharova M, Babak V (2009). Monitoring major mastitis pathogens at the population level based on examination of bulk tank milk samples. J. Dairy Res.

[11] Zanardi G, Caminiti A, Delle DG, Moroni P, Santi A, Galletti G, Tamba M, Bolzoni G, Bertocchi L (2014). Short communication: Comparing real-time PCR and bacteriological cultures for *Streptococcus agalactiae* and *Staphylococcus aureus* in bulk-tank milk samples. J. Dairy Sci.

[12] Ronco T, Klaas IC, Stegger M, Svennesen L, Astrup LB, Farre M, Pedersen K (2018). Genomic investigation of *Staphylococcus aureus* isolates from bulk tank milk and dairy cows with clinical mastitis. Vet. Microbiol.

[13] Ruegg PL (2017). A 100-year review: Mastitis detection, management, and prevention. J. Dairy Sci.

[14] Down PM, Bradley AJ, Breen JE, Hudson CD, Green MJ (2016). Current management practices and interventions prioritised as part of a nationwide mastitis control plan. Vet. Rec.

[15] Petzer IM, Karzis J, Donkin EF, Webb EC (2016). A pathogen-specific approach towards udder health management in dairy herds: Using culture and somatic cell counts from routine herd investigations. Onderstepoort J. Vet. Res.

[16] Rodrigues AC, Caraviello DZ, Ruegg PL (2005). Management of wisconsin dairy herds enrolled in milk quality teams. J. Dairy Sci.

[17] Birhanu M, Leta S, Mamo G, Tesfaye S (2017). Prevalence of bovine subclinical mastitis and isolation of its major causes in Bishoftu Town, Ethiopia. BMC Res. Notes.

[18] Hajna AA, Perry C.A (1943). Comparative study of presumptive and confirmative media for bacteria of the coliform group and for fecal streptococci. Am. J. Public Health Nations Health.

[19] Hogan JS, Gonzalez RN, Harmon RJ, Nickerson SC, Oliver SP, Pankey JW, Smith KL (1999). Laboratory Handbook on Bovine Mastitis.

[20] Yang FY, Shen C, He BX, Yang YY, Gong DC, Li XS (2015). The prevalence of heifer mastitis and its associated risk factors in Huanggang, Central China. Trop. Anim. Health Prod.

[21] Bramley AJ, McKinnon CH, Staker RT, Simpkin DL (1984). The effect of udder infection on the bacterial flora of the bulk milk of ten dairy herds. J. Appl. Bacteriol.

[22] Godkin MA, Leslie KE (1993). Culture of bulk tank milk as a mastitis screening test: A brief review. Can. Vet. J.

[23] Jayarao BM, Wolfgang DR (2003). Bulk-tank milk analysis. A useful tool for improving milk quality and herd udder health. Vet. Clin. N. Am. Food Anim. Pract.

[24] Hand KJ, Godkin A, Kelton DF (2012). Milk production and somatic cell counts: A cow-level analysis. J. Dairy Sci.

[25] Sharma N, Singh NK, Bhadwal MS (2011). Relationship of somatic cell count and mastitis: An overview. Asian Aust. J. Anim. Sci.

